# Accuracy of mobile 6-lead electrocardiogram device for assessment of QT interval: a prospective validation study

**DOI:** 10.1007/s12471-022-01716-5

**Published:** 2022-09-05

**Authors:** A. T. Bergeman, S. N. J. Pultoo, M. M. Winter, G. A. Somsen, I. I. Tulevski, A. A. M. Wilde, P. G. Postema, C. van der Werf

**Affiliations:** 1 Department of Clinical and Experimental Cardiology, Heart Centre, Amsterdam Cardiovascular Sciences, Amsterdam University Medical Centres, location Academic Medical Centre, University of Amsterdam, Amsterdam, The Netherlands; 2 Cardiology Centres of the Netherlands, Amsterdam, The Netherlands

**Keywords:** QT interval, Telehealth, Electrocardiography, Long QT syndrome, Sudden cardiac death

## Abstract

**Introduction:**

Ambulatory assessment of the heart rate–corrected QT interval (QTc) can be of diagnostic value, for example in patients on QTc-prolonging medication. Repeating sequential 12-lead electrocardiograms (ECGs) to monitor the QTc is cumbersome, but mobile ECG (mECG) devices can potentially solve this problem. As the accuracy of single-lead mECG devices is reportedly variable, a multilead mECG device may be more accurate.

**Methods:**

This prospective dual-centre study included outpatients visiting our cardiology clinics for any indication. Participants underwent an mECG recording using a smartphone-enabled 6‑lead mECG device immediately before or immediately after a conventional 12-lead ECG recording. Multiple QTc values in both recordings were manually measured in leads I and II using the tangent method and subsequently compared.

**Results:**

In total, 234 subjects were included (mean ± standard deviation (SD) age: 57 ± 17 years; 58% males), of whom 133 (57%) had cardiac disease. QTc measurement in any lead was impossible due to artefacts in 16 mECGs (7%) and no 12-lead ECGs. Mean (± SD) QTc in lead II on the mECG and 12-lead ECG was 401 ± 30 and 406 ± 31 ms, respectively. Mean (± SD) absolute difference in QTc values between both modalities was 12 ± 9 ms (r = 0.856; *p* < 0.001). In 55% of the subjects, the absolute difference between QTc values was < 10 ms.

**Conclusion:**

A 6-lead mECG allows for QTc assessment with good accuracy and can be used safely in ambulatory QTc monitoring. This may improve patient satisfaction and reduce healthcare costs.

## What’s new?

- In a general cardiology outpatient population, a 6-lead mobile electrocardiogram (mECG) device allowed for accurate measurement of the heart rate–corrected QT interval (QTc), with high interobserver reliability.
- mECG QTc measurements in lead II showed better agreement with the 12-lead ECG QTc measurements than those in lead I.
- Although significant movement artefacts occurred in a substantial proportion of 6‑lead mECGs, the QTc could be measured in 93% of the recordings.

## Introduction

An abnormally shortened or prolonged heart rate–corrected QT interval (QTc) predisposes to potentially life-threatening ventricular tachyarrhythmias [1, 2]. Hence, QTc monitoring is of vital importance in patients at risk of arrhythmia due to QTc prolongation. Assessment of the QTc is traditionally performed using a 12-lead electrocardiogram (ECG), but this type of recording requires a visit to a cardiology outpatient clinic or other medical facility.

Mobile ECG (mECG) devices can potentially simplify QTc monitoring and be more cost-effective than utilising 12-lead ECG recorders. The infrastructure of existing remote arrhythmia monitoring programmes could facilitate the introduction of QTc monitoring if measurements are sufficiently reliable. The available literature on the utility of mECGs for measuring the QTc mostly describes single-lead devices and shows mixed results [3]. Single-lead mECGs often mimic lead I, whereas other leads, such as lead II, are conventionally used to determine the QTc on 12-lead ECGs [4]. Therefore, a multilead mECG may enable more accurate measurement. Currently, only a few studies have directly compared the accuracy of determining the QTc on a 6-lead mECG [5–7]. The objective of this study was to assess the accuracy of a 6-lead mECG device in measuring the QTc in a general cardiology outpatient population.

## Methods

### Study population

Individuals visiting the outpatient clinics of the Cardiology Centres of the Netherlands or the Amsterdam University Medical Centres, location Academic Medical Centre between December 2020 and May 2021 with an indication for a 12-lead ECG were invited to participate and were included in the study after obtaining their written informed consent. Those who were physically unable to use the mECG device or could not provide informed consent were not invited to participate. The Medical Research Ethics Committee of the Amsterdam University Medical Centres, location Academic Medical Centre approved this study.

### Data collection

Six-lead mECG recordings were obtained using the KardiaMobile 6L (Alivecor Inc., Mountain View, CA, USA), which is a small (9.0 × 3.0 × 0.72 cm), wireless mECG device that can directly record leads I and II and derive leads III, aVL, aVF and aVR. This device consists of three electrodes: two on the top surface for both thumbs and one on the bottom surface, which makes contact with either the left ankle or left knee. Through Bluetooth, the device connects to the corresponding application on smartphones and tablets to record a 30-second 6‑lead mECG. It provides an automated assessment of the heart rhythm and heart rate. At present, the KardiaMobile 6L is the only commercially available multilead mECG device to our knowledge.

All subjects underwent conventional 12-lead ECG recording whilst supine. Within 5 min before or after this recording, a 30-second mECG tracing was recorded in sitting position. Depending on what was most convenient to the participant, either the left ankle or left knee was used.

### Measurements

Heart rate, PR interval, QRS duration and QT interval were manually assessed by one reader (AB) on all mECG recordings and 12-lead ECGs. Additionally, the heart rhythm as well as the automated algorithmic rhythm assessment of the mECGs were documented. All recordings were analysed in digital format and measurements were performed using EP Calipers software (EP Studios, Inc., Louisville, KY, USA). All recordings were analysed in an unblinded but random order to avoid consecutive assessment of the mECG and 12-lead ECG recordings of the same participant.

**Fig. 1:**
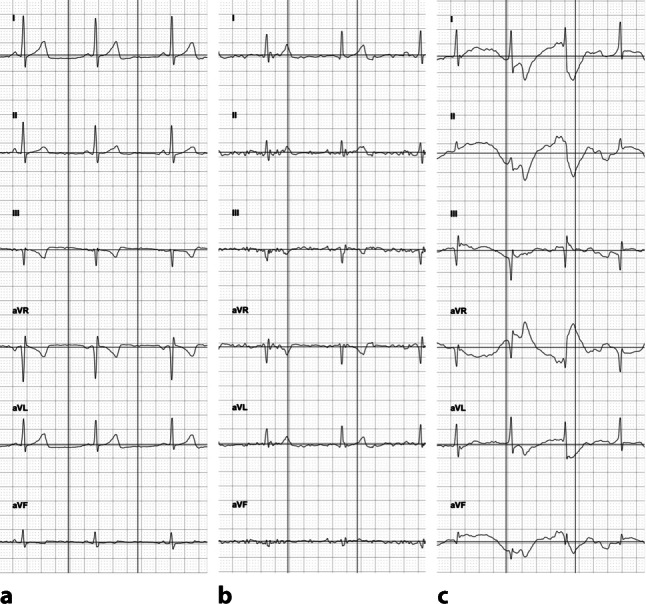
Representative mobile electrocardiograms illustrating quality of the recording. **a** Good quality. **b** Acceptable quality. **c** Poor quality

The quality of the mECG recordings was classified as ‘good’, ‘acceptable’ or ‘poor’ (Fig. 1). Recordings with no or minimal artefacts were categorised as being of good quality. Acceptable quality was defined as a recording in which the QT interval could be measured but significant artefacts were present, limiting reliable identification of atrial activity. In recordings of poor quality, the QT interval could not be measured in any lead.

On all mECG and 12-lead recordings, three QT intervals were measured manually in both leads I and II using the tangent method [4]. The three QT intervals with the least artefacts were chosen. If it was not possible to measure three complexes due to significant artefacts, ≤ 2 complexes were chosen. On the mECG recordings, the selected QT intervals were often nonconsecutive. To calculate the beat-level QTc using the preceding RR interval, Bazett’s formula was used, after which the mean of the three QTc values was calculated.

To assess interobserver reliability of QTc measurements, a second reader (SP) measured the mean QTc of a randomly chosen subset (10%) of recorded mECGs and 12-lead ECGs in similar fashion. In this subset, a cardiologist (CvdW) marked three complexes, which were then measured by the two readers to avoid differences in QT/QTc due to measurement of different RR-QT complexes. If it was not possible to mark three complexes, ≤ 2 complexes were chosen.

### Statistical analysis

Categorical variables are expressed as frequencies and percentages, and continuous variables are expressed as mean ± standard deviation (SD) for normal distributions. Pearson correlation analysis was used to examine the relationship between heart rate, PR interval, QRS duration and QTc values on the 12-lead ECG versus the 6‑lead mECG recordings. The Bland-Altman method of analysis of measurement agreement was employed to determine the agreement in QTc measurements between the mECG and 12-lead ECG recordings, with the QTc value on the 12-lead ECG being considered the reference when calculating the mean difference and the 95% limits of agreement. We defined perfect agreement as an absolute difference in QTc values between both modalities < 10 ms. Intraclass correlation coefficients were used to assess the degree of interobserver reliability of QTc measurements. Finally, the sensitivity and specificity of identifying a prolonged QTc, defined as QTc ≥ 480 ms [2], on the mECG as compared with the 12-lead ECG were calculated.

Statistical analysis was performed using IBM SPSS Statistics, version 27 (IBM Corp., Armonk, NY, USA). A two-tailed probability value of < 0.05 was considered statistically significant.

## Results

In total, 235 subjects agreed to participate and underwent both an mECG and 12-lead ECG recording; one subject later withdrew consent. Mean age was 57 ± 17 years (range: 18–90), 136 subjects (58%) were male, and 133 participants (57%) had a history of cardiac disease (Tab. 1).

**Table 1:** Participant characteristics

Variable	Participants (*N* = 234)
Male	136 (58.1)
Age, years	56.9 ± 17.2
BMI, kg/m^2^	26.3 ± 4.4
Use of QTc-prolonging medication	27 (11.5)
*Noncardiac medical history*	130 (55.6)
Hypertension	89 (38.0)
Dyslipidaemia	70 (29.9)
Diabetes mellitus	27 (11.5)
Pulmonary embolism/DVT	9 (3.8)
Stroke/TIA	18 (7.7)
*Cardiac disease*	133 (56.8)
Atrial fibrillation	38 (16.2)
Heart failure	15 (6.4)
Stable coronary artery disease	46 (19.7)
Acute coronary syndrome	35 (15.0)
Valvular heart disease	35 (15.0)
Congenital long QT syndrome	10 (4.3)

Data are *n* (%) or mean ± standard deviation

*BMI* body mass index, *QTc* heart rate–corrected QT interval, *DVT* deep venous thrombosis, *TIA* transient ischaemic attack

Sixteen recordings (7%) were of poor quality. Of the remaining mECGs, 74 (32%) and 144 (62%) were of good and acceptable quality, respectively, indicating that significant artefacts were present in approximately two-thirds of mECGs.

At the time of ECG acquisition, 217 subjects (93%) were in sinus rhythm, 13 (6%) were in atrial fibrillation, and 4 (2%) showed another rhythm. Of the 234 mECG recordings, 215 (92%) were determined to show sinus rhythm and 15 (6%) indicated the presence of atrial fibrillation. One mECG (0.4%) was interpreted as showing an atrial rhythm, whereas the remaining three mECGs were of insufficient quality to determine the heart rhythm. There was agreement between the manual mECG rhythm assessment and 12-lead ECG rhythm in 227 of 231 subjects (98%).

The comparison of the heart rate and conduction interval measurements is summarised in Tab. 2. Heart rate on the mECG was consistently higher than that on the 12-lead ECG (mean difference: 9 ± 8 bpm; *p* < 0.001). Comparisons of the PR interval and QRS duration using both modalities showed a moderate to strong degree of correlation (r = 0.763 and r = 0.639, respectively). The PR interval could not be determined in two sinus rhythm mECG recordings, while the QRS duration could not be determined in one mECG recording.

**Table 2:** Comparison of heart rate and interval measurements on 12-lead ECG and 6‑lead mECG

	12-lead ECG		mECG				
Variable	*N*	Mean ± SD	*N*	Mean ± SD	Mean difference ± SD	Mean absolute difference ± SD	Pearson correlation coefficient
Heart rate, bpm	234	69 ± 15	233	78 ± 17	9 ± 8	10 ± 7	0.882
PR interval, ms	217	167 ± 29	213	161 ± 28	−5 ± 20	16 ± 13	0.763
QRS duration, ms	234	99 ± 16	233	97 ± 17	−3 ± 14	11 ± 8	0.639
QTc (lead I), ms	191	395 ± 30	201	396 ± 30	1 ± 19	14 ± 13	0.783
QTc (lead II), ms	231	406 ± 31	203	401 ± 30	−3 ± 16	12 ± 9	0.856

*ECG* electrocardiogram; *mECG* mobile electrocardiogram; *SD* standard deviation; *QTc* heart rate–corrected QT interval

### Comparison of QTc measurements

In a subset of ECGs, it was not possible to measure the QTc, either due to artefacts or low amplitudes. Of the 234 mECG recordings, 33 recordings did not allow for QTc measurement in lead I and 31 did not allow for QTc measurement in lead II. Neither lead was suitable in 16 mECGs. As for 12-lead ECGs, QTc measurement was not possible in leads I and II in 43 and 3 recordings, respectively. One 12-lead ECG did not allow for QTc measurement in either lead.

**Fig. 2:**
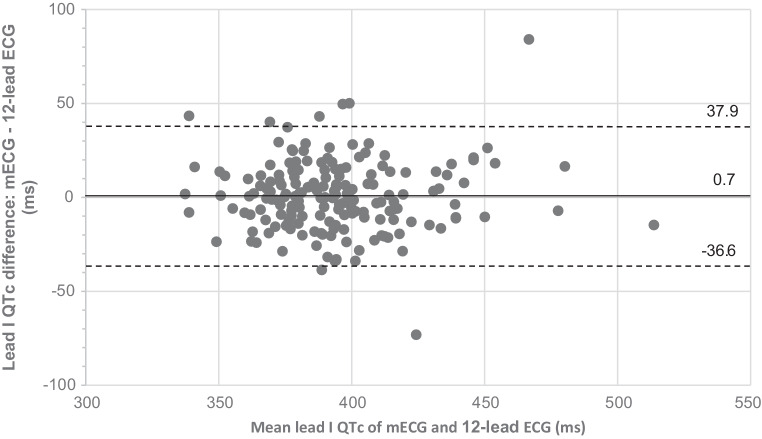
Bland-Altman plot showing measurement agreement of QTc values between 6‑lead mobile electrocardiogram (*mECG*) and 12-lead electrocardiogram (*ECG*) recordings in lead I (*n* = 177) (*middle solid line* represents overall mean difference, whereas *dotted lines* show 95% limits of agreement)

When comparing QTc in lead I, overall mean QTc on mECG (*n* = 201) and 12-lead ECG (*n* = 191) was 396 ± 30 ms and 395 ± 30 ms, respectively (*p* = 0.839). The mean absolute difference in QTc values between the modalities using lead I was 14 ± 13 ms (r = 0.783; *p* < 0.001); the absolute difference was < 10 ms in 78 recordings (44%) (Tab. 2). The degree of measurement agreement in lead I was acceptable (Fig. 2).

**Fig. 3:**
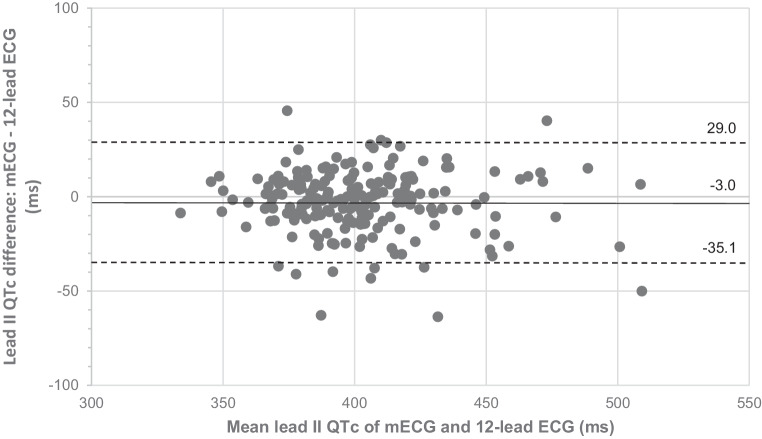
Bland-Altman plot showing measurement agreement of QTc values between 6‑lead mobile electrocardiogram (*mECG*) and 12-lead electrocardiogram (*ECG*) recordings in lead II (*n* = 203) (*middle solid line* represents overall mean difference, whereas *dotted lines* show 95% limits of agreement)

Using lead II measurements, mean QTc on the mECG (*n* = 203) was shorter than that on the 12-lead ECG (*n* = 231): 401 ± 30 versus 406 ± 31 ms (*p* = 0.038) (Tab. 2). The mean absolute difference in lead II QTc between the modalities was 12 ± 9 ms, which corresponded with a strong correlation (r = 0.856, *p* < 0.001). In 112 subjects (55%), the absolute difference was < 10 ms, which meant there was perfect agreement. Lead II mECG and 12-lead ECG QTc measurements showed good agreement using Bland-Altman analysis (Fig. 3).

Six subjects had a mean QTc ≥ 480 ms in lead II on the 12-lead ECG. The sensitivity and specificity for lead II mECG QTc prolongation were 80% and 99%, respectively (*n* = 203).

When comparing the mECG QTc measurements performed by the two interpreters, the intraclass correlation coefficients were 0.976 for lead I and 0.952 for lead II. For 12-lead ECG QTc measurements, the intraclass correlation coefficients were 0.958 for lead I and 0.965 for lead II.

## Discussion

This prospective study in a representative population of cardiology outpatients showed a good degree of accuracy of QTc measurements between 6‑lead mECG and 12-lead ECG recordings. Lead II was slightly more accurate than lead I, although lead II did underestimate the QTc marginally, albeit significantly. Other conduction interval measurements showed comparable levels of agreement. We found that the level of interobserver variability for QTc measurements using the mECG was low, similar to the findings when analysing the 12-lead ECG recordings.

The present study is among the first to assess the accuracy of a 6-lead mECG of measuring the QTc. Kleiman et al. conducted a study with a similar design in a large cohort of patients (*n* = 705) presenting at a genetic arrhythmia clinic [5]. They found a mean difference of −2.6 ms between 6‑lead mECG and 12-lead ECG QTc values, as well as an absolute difference smaller than 10 ms in 44% of the patients. The principal distinction with our study is the patient population: our study included a sample of a general cardiology outpatient population who were older and had more comorbidities.

Azram et al. performed a large validation study of the 6‑lead mECG in both cardiology outpatients and inpatients [6]. Their results showed that roughly 20% of the mECGs did not allow for QTc measurements. The mean difference between 6‑lead mECG and 12-lead ECG QTc values was −0.27 ± 28.17 ms (lead I) and 0.62 ± 26.82 ms (lead II), indicating that the difference between QTc values was more dispersed compared with our findings. Surprisingly, the level of agreement was higher for the uncorrected QT values. Another similar, albeit smaller study performed in 30 athletes by Orchard et al. found a comparable but slightly larger mean difference in QTc values between 6‑lead mECG and 12-lead ECG [7].

Giudicessi et al. recently showed that an artificial intelligence algorithm is able to accurately determine the QTc when applied to 6‑lead mECG recordings in a large cohort of patients [8]. In their study, however, the accuracy of the 6‑lead mECG was not directly compared with that of the 12-lead ECG. Studies using single-lead mECG measurements of the QTc consistently found an inferior degree of accuracy [9–11], suggesting that a multilead mECG may indeed be more reliable.

We found that the heart rate was consistently higher on mECG tracings than on 12-lead ECG recordings. This is attributable to the sequential order of recordings: in most subjects, the 12-lead ECG was recorded in a supine position and the mECG in a sitting position directly before or afterwards, which caused an increase in heart rate. Because the various QT interval correction formulae are imperfect, this likely had an undue effect on QTc values of unknown significance. Even when disregarding the effects of heart rate, positional changes can alter the duration of the QT interval, particularly in patients with congenital long QT syndrome [12]. In two of the previously mentioned studies, this heart rate difference was still present, albeit less pronounced, due to a longer time interval between the recordings [5, 7]. Azram et al. did not report on heart rate comparisons [6]. Although a longer time between the two recordings and a subsequent smaller heart rate difference might seem favourable for comparing the QTc, the odds of observing diverging QT intervals due to spontaneous QTc variability increases. From this perspective, recording both tracings consecutively is a strong point of our study.

mECG devices offer unique advantages over traditional 12-lead ECG recorders. Firstly, an ECG can be recorded with an mECG device at any location. The simplicity of use also allows operators without experience to record an ECG nearly instantaneously. Secondly, the mECG device does not utilise any disposables, thereby limiting waste and reducing the carbon footprint. Lastly, its low cost of use renders the device usable in a wide range of settings and could make QTc monitoring more cost-effective. Multilead mECG devices may be used to remotely monitor the QTc by having patients at risk of QTc prolongation sending mECGs to their cardiologist at predetermined moments, thereby potentially mitigating a visit to the outpatient clinic or hospital. Another advantage is the ability to perform repeated QTc measurements on multiple days.

### Study limitation

A limitation of our study is that significant artefacts complicated the interpretation of the majority of mECGs. Several mECGs (7%) were even uninterpretable. This emphasises the need to better instruct subjects to sit still during the 30-second recording, which was often difficult for elderly individuals. Improvements in noise filtering are warranted, although this may distort relatively low frequency signals such as the TU-complex.

## Conclusion

The use of a 6-lead mECG enables QTc measurement with a good degree of accuracy compared with 12-lead ECG. As QTc measurement in lead II was more accurate than that in lead I, it is advisable to use lead II of the 6‑lead mECG. In a general cardiology outpatient population, 6‑lead mECG can be used for remote monitoring of QTc in individuals who are at risk of QTc prolongation and subsequent arrhythmia. Remote QTc assessment may improve patient satisfaction and safety and contribute to cost-effectiveness in cardiology care.

## References

[1] Shah SR, Park K, Alweis R (2019). Long QT syndrome: A comprehensive review of the literature and current evidence. Curr Probl Cardiol.

[2] Priori SG, Blomström-Lundqvist C, Mazzanti A (2015). 2015 ESC Guidelines for the management of patients with ventricular arrhythmias and the prevention of sudden cardiac death: The Task Force for the Management of Patients with Ventricular Arrhythmias and the Prevention of Sudden Cardiac Death of the European Society of Cardiology (ESC). Endorsed by: Association for European Paediatric and Congenital Cardiology (AEPC). Eur Heart J.

[3] Bansal A, Joshi R (2018). Portable out-of-hospital electrocardiography: A review of current technologies. J Arrhythm.

[4] Postema PG, Wilde AA (2014). The measurement of the QT interval. Curr Cardiol Rev.

[5] Kleiman R, Darpo B, Brown R (2021). Comparison of electrocardiograms (ECG) waveforms and centralized ECG measurements between a simple 6-lead mobile ECG device and a standard 12-lead ECG. Ann Noninvasive Electrocardiol.

[6] Azram M, Ahmed N, Leese L (2021). Clinical validation and evaluation of a novel six-lead handheld electrocardiogram recorder compared to the 12-lead electrocardiogram in unselected cardiology patients (EVALECG Cardio). Eur Heart J Digit Health.

[7] Orchard JJ, Orchard JW, Raju H, La Gerche A, Puranik R, Semsarian C (2021). Comparison between a 6-lead smartphone ECG and 12-lead ECG in athletes. J Electrocardiol.

[8] Giudicessi JR, Schram M, Bos JM (2021). Artificial intelligence-enabled assessment of the heart rate corrected QT interval using a mobile electrocardiogram device. Circulation.

[9] Haberman ZC, Jahn RT, Bose R (2015). Wireless smartphone ECG enables large-scale screening in diverse populations. J Cardiovasc Electrophysiol.

[10] Cheung CC, Davies B, Gibbs K, Laksman ZW, Krahn AD (2020). Multilead QT screening is necessary for QT measurement: Implications for management of patients in the COVID-19 era. JACC Clin Electrophysiol.

[11] Bekker CL, Noordergraaf F, Teerenstra S, Pop G, van den Bemt BJF (2020). Diagnostic accuracy of a single-lead portable ECG device for measuring QTc prolongation. Ann Noninvasive Electrocardiol.

[12] Viskin S, Postema PG, Bhuiyan ZA (2010). The response of the QT interval to the brief tachycardia provoked by standing: a bedside test for diagnosing long QT syndrome. J Am Coll Cardiol.

